# Monocyte chemoattractant protein-1 (MCP-1)-2518 A/G polymorphism and lupus nephritis risk: A PRISMA-compliant meta-analysis

**DOI:** 10.1097/MD.0000000000009401

**Published:** 2017-12-22

**Authors:** Guo-Yao Sang, Cun-Ren Meng, Yun-Fei Hao, Jiang-Hong Dai

**Affiliations:** a Laboratory Medicine Diagnostic Centre; b Periodontal and mucosal department, The First Affiliated Hospital; c Department of Epidemiology and biostatistics, School of Public Health, Xinjiang Medical University, Urumqi, Xinjiang, China.

**Keywords:** lupus nephritis, meta-analysis, monocyte chemoattractant protein-1, polymorphism

## Abstract

**Background:**

Monocyte chemoattractant protein-1 (MCP-1) plays an important role in the development of allergic inflammatory reactions by recruiting various immune cells, which is associated with many autoimmune diseases, but the association with the MCP-1-2518A/G gene polymorphism and lupus nephritis (LN) was still controversial in previous studies. Thus, we performed a meta-analysis to derive a more precise evaluation of the association between MCP-1 -2518A/G polymorphism and LN risk and evaluated influence of ethnicity and source of controls.

**Methods:**

A systematic review and meta-analysis that will be performed according to the Preferred Reporting Items for Systematic Reviews and Meta-Analyses (PRISMA). Relevant literatures dated to September 2016 were acquired from the PubMed, EMBASE, Cochran Library databases. A total of 961 LN cases and 1867 controls were extracted from 10 published case-control studies. We used odds ratios (OR) with 95% confidence intervals (CI) to assess the risk of LN with MCP-1-2518A/G.

**Results:**

Our meta-analysis suggested that MCP-1-2518A/G polymorphism was associated with the risk of LN (GG vs AG+AA: *P* < .01, OR = 1.42, 95% CI: 1.13–1.79 and A vs G *P* = .02, OR = 0.74, 95% CI: 0.58–0.95). Then the subgroup analysis showed MCP-1 -2518 A/G gene has a certain correlation with LN susceptibility in the American population (GG vs AA: *P* < .01, OR = 5.70, 95% CI: 2.09–15.50, GG vs AG+AA: *P* < .01, OR = 3.31, 95% CI: 1.97–5.54, GG+AG vs AA: *P* < .01, OR = 2.86, 95% CI: 1.14–7.18, and A vs G: *P* < .01, OR = 0.43, 95% CI: 0.24–0.79), while no significant risk in Europeans and Asians.

**Conclusion:**

The current meta-analysis suggests that the MCP-1-2518A/G polymorphism is associated with an increased risk of LN, especially in the American population. However, better-designed studies with larger sample sizes are needed to validate the results.

## 1 Introduction

Lupus nephritis (LN) is a result of systemic lupus erythematosus (SLE), as one of the most serious complications of SLE, which leads to the deposition of glomerular immune complex and the inflammation of renal glomerular injury.^[1]^ Previous epidemiological studies suggested LN had obvious familial aggregation which was closely related to many factors, such as multiple susceptibility genes and mRNA.^[2]^ With further investigation, the genetic variation of copy number variation and epigenetic changes were confirmed to be associated with the incidence of LN.^[3]^

Monocyte chemoattractant protein-1 (MCP-1) plays an important role in the development of allergic inflammatory reactions by recruiting various immune cells, which is associated with diverse autoimmune diseases.^[4]^ In addition, Singh et al^[5]^ found the level of urinary MCP-1 in LN patients was significantly higher than those without LN, and correlated to the severity of LN, which indicated MCP-1 might be a biological indicator of LN. Subsequently, Mohammed's research^[6]^ further confirmed the association of MCP-1 and susceptibility LN. However, Alharazy et al^[7]^ noted that there was no certain association between the level of MCP-1 and the LN. Furthermore, the gene phenotype of MCP-1 -2518A/G was not associated with the incidence of LN.^[8]^ Obviously, the association with the MCP-1-2518A/G gene polymorphism and LN was still controversial. Considering the relatively small sample size in most studies, it is possible to perform a quantitative synthesis of the evidence with rigorous methods. Here, we performed a meta-analysis on 10 published case-controls to derive a more precise evaluation of the association between MCP-1-2518A/G polymorphism and LN risk.

## 2 Materials and methods

### 2.1 Bibliographic search

The keywords “monocyte chemoattractant protein-1,” “MCP-1,” “lupus nephritis,” “systemic lupus erythematosus,” and “polymorphism” were searched in 3 databases (PubMed, Embase, Cochrane Library) for entries until September 2016. References of the retrieved publications were also screened. All eligible studies were retrieved, and their bibliographies were checked for other relevant publications. Only published studies with full-text articles were included. When overlapping articles were found, we only included the publications that reported the most extensive information.

### 2.2 Inclusion criteria

Literatures fulfilled the following criteria: published in English; experimental subjects were the LN patients, and the control groups were healthy people; case-control studies of LN with MCP-1-2518 A/G polymorphism; sufficient published data for estimating an odds ratio (OR) with 95% confidence interval (CI); case control groups genotype conformed to the Hardy Weinberg (H-W) balance.

### 2.3 Data extraction

Two investigators independently reviewed the articles to exclude irrelevant and overlapping studies. The results were compared, and disagreements were resolved by discussion and consensus. We extracted the following information from each study: first author's surname, publish year, ethnicity, and the number of cases and controls for each genotype, gene detection method, and control source.

### 2.4 Statistical analysis

The odds ratio (OR) with 95% confidence interval (CI) was used to assess the strength of association between MCP-1-2518 A/G polymorphisms and LN risk in 5 genetic models (GG vs AA, GG vs AG+AA, GG+GA vs AA, AG vs AA, and A vs G). The *χ*^2^-based Q statistic test was performed to evaluate the between-study heterogeneity of studies. If the heterogeneity was not significant (*P* > .1, I^2^<50.0%), then the fixed-effect model can be performed, otherwise, the random-effect model. Subgroup analyses were conducted among variables, such as ethnicities and source of controls. Sensitivity analysis was conducted by removing 1 data set at a time to identify individual study's effect on pooled results and test the reliability of results. Funnel plots were used to access the potential publication bias by the method of Egger linear regression test. All analyses were performed by Stata (version 12.0, Stata Corporation) and Review Manager (version 5.0.0, The Cochrane collaboration), using 2 side *P* values.

## 3 Results

### 3.1 Search results

Our search strategy identified 34 potentially relevant studies, and a total of 10 literatures^[8–17]^ were adopted in the end, including 961 LN cases and 1867 controls met the including criteria. The populations were from the Americas, Europe, and Asia. A classic polymerase chain reaction–restriction fragment length polymorphism assay was performed in all studies. The characteristics of these studies and the quality scores were shown in Table 1.

**Table 1:**
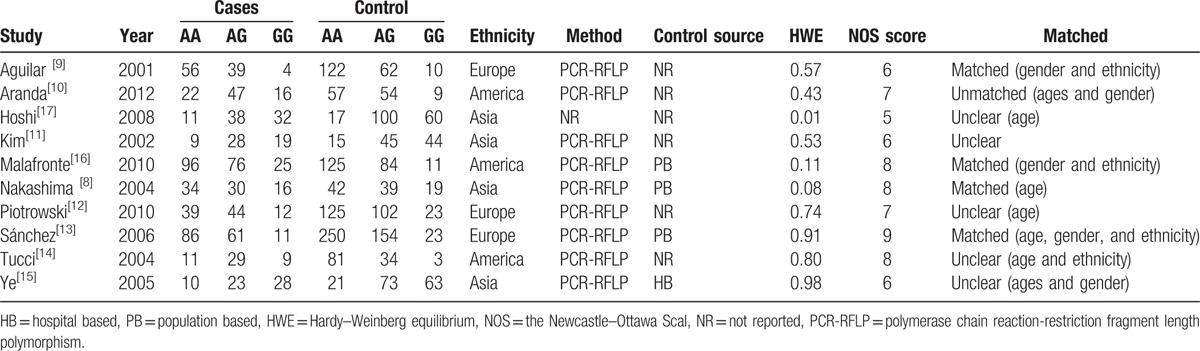
Characteristics of published studies included in this meta-analysis.

### 3.2 Main results

**Figure 1:**
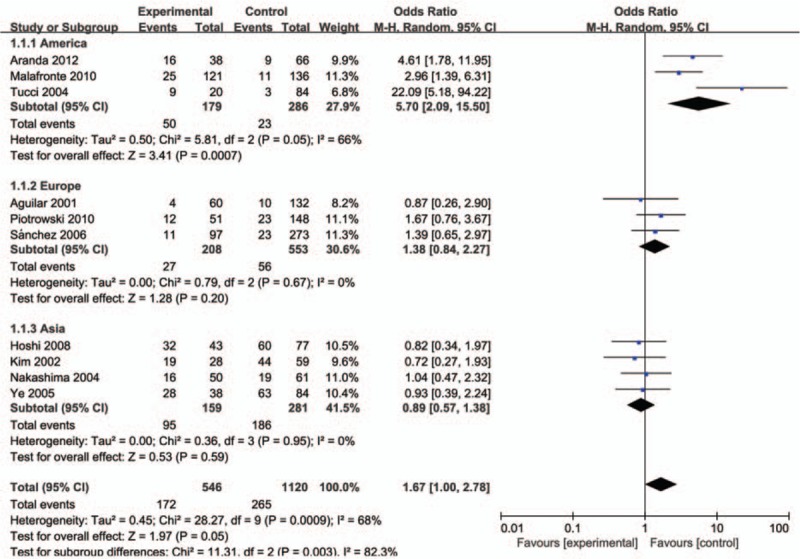
Meta-analysis for the OR of LN associated with MCP-1-2518 A/G polymorphism (GG vs AA). LN = lupus nephritis, MCP-1 = monocyte chemoattractant protein-1, OR = odds ratios.

**Figure 2:**
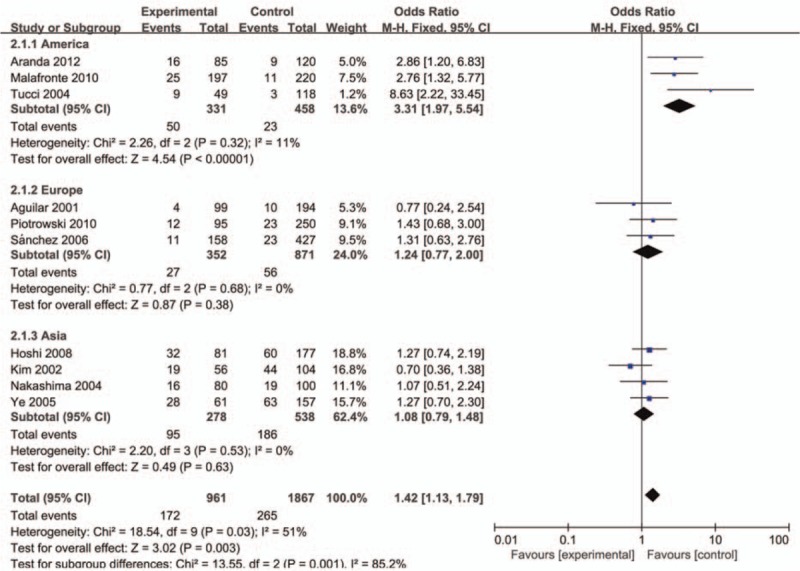
Meta-analysis for the OR of LN associated with MCP-1-2518 A/G polymorphism (GG vs AA+AG). LN = lupus nephritis, MCP-1 = monocyte chemoattractant protein-1, OR = odds ratios.

**Figure 3:**
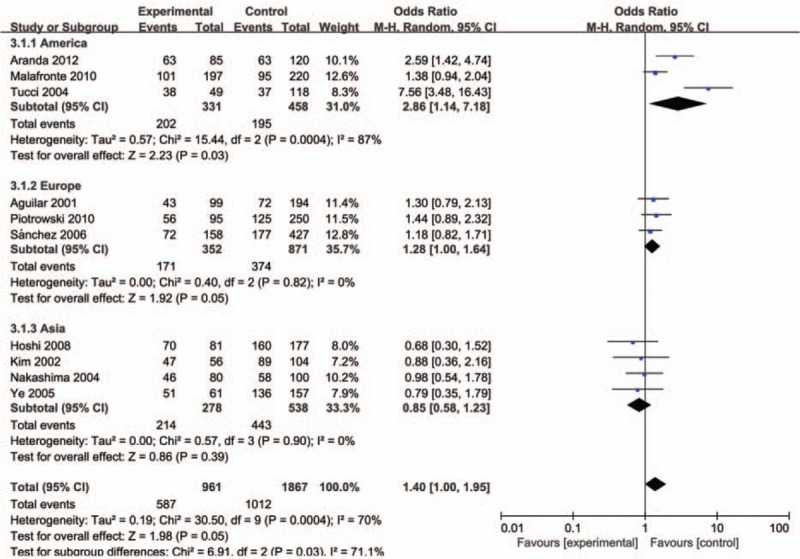
Meta-analysis for the OR of LN associated with MCP-1-2518 A/G polymorphism (GA+GG vs AA). LN = lupus nephritis, MCP-1 = monocyte chemoattractant protein-1, OR = odds ratios.

**Figure 4:**
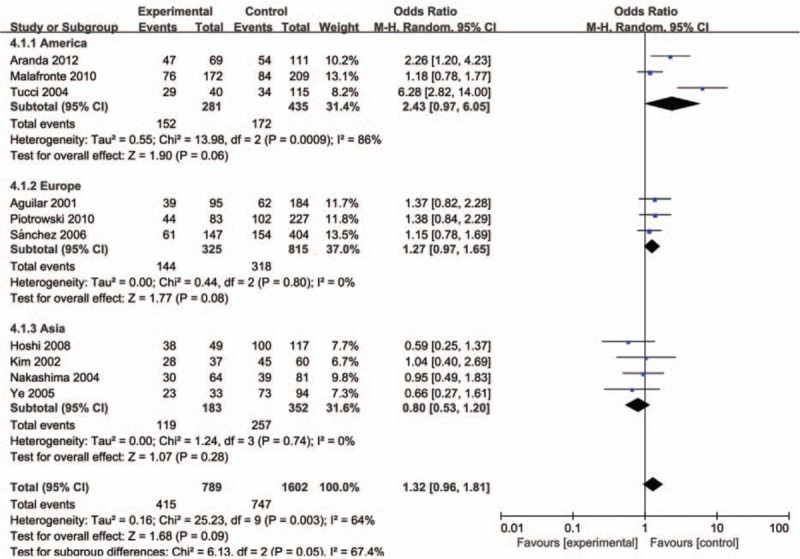
Meta-analysis for the OR of LN associated with MCP-1-2518 A/G polymorphism (AG vs. AA). LN = lupus nephritis, MCP-1 = monocyte chemoattractant protein-1, OR = odds ratios.

**Figure 5:**
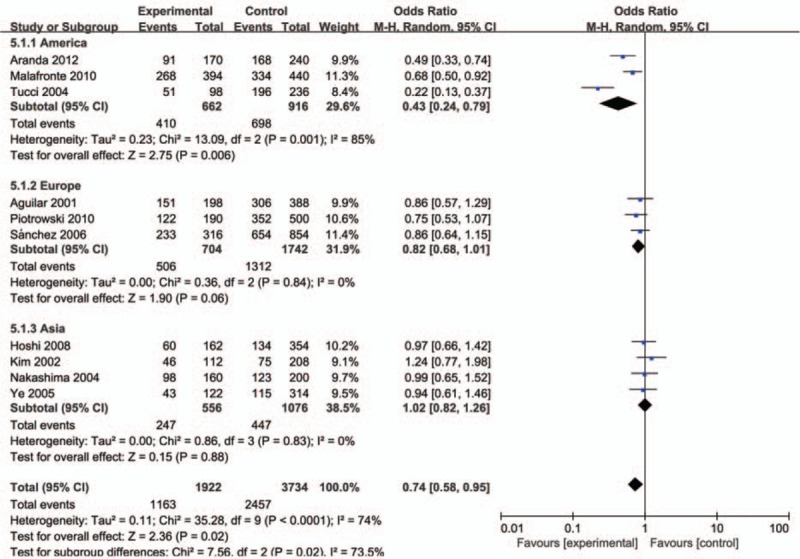
Meta-analysis for the OR of LN associated with MCP-1-2518 A/G polymorphism (A vs G). LN = lupus nephritis, MCP-1 = monocyte chemoattractant protein-1, OR = odds ratios.

The evaluation of association between MCP-1-2518A/G polymorphism and LN risk was presented in Table 2. Overall, there was correlation between the susceptibility of MCP-1 gene and LN, and the difference has statistical significance (GG vs AG+AA: *P* = .00, OR = 1.42, 95% CI: 1.13–1.79 and A vs G: *P* = .02, OR = 0.74, 95% CI: 0.58–0.95, respectively). Subsequently, subgroup analysis was performed based on the ethnicity (the Americas, Europe, and Asia), which suggested the MCP-1-2518 A/G gene has a certain correlation with LN susceptibility in the American population GG vs AA: *P* < .01, OR = 5.70, 95% CI: 2.09–15.50, GG vs AG+AA: *P* < .01, OR = 3.31, 95% CI: 1.97–5.54, GG+AG vs AA: *P* < .01, OR = 2.86, 95% CI: 1.14–7.18, and A vs G: *P* < .01, OR = 0.43, 95% CI: 0.24–0.79; Figs. 1–5), while no significant risk in Europeans and Asians. When stratified according to sources of controls, we found significant main effects for MCP-1 -2518 A/G polymorphism on LN risk in the population based sources of controls (GG vs AA+AG: OR:1.60, 95% CI: 1.05–2.43).

**Table 2:**
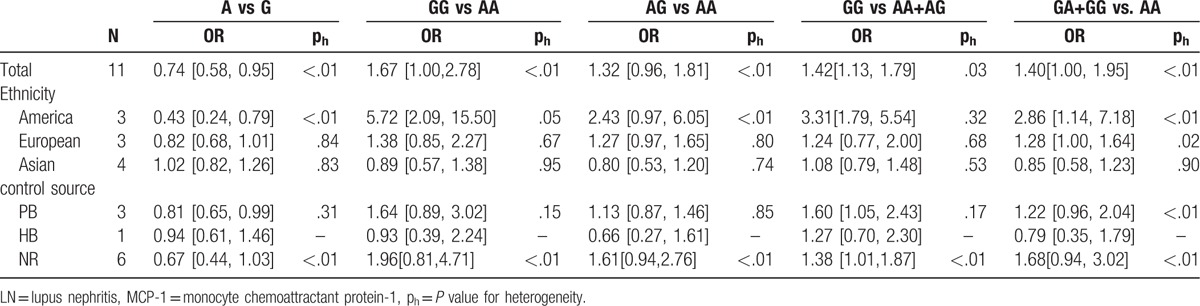
Total and stratified analyses of the MCP-1-2518 A/G polymorphism on LN risk.

### 3.3 Sensitivity analysis

**Figure 6:**
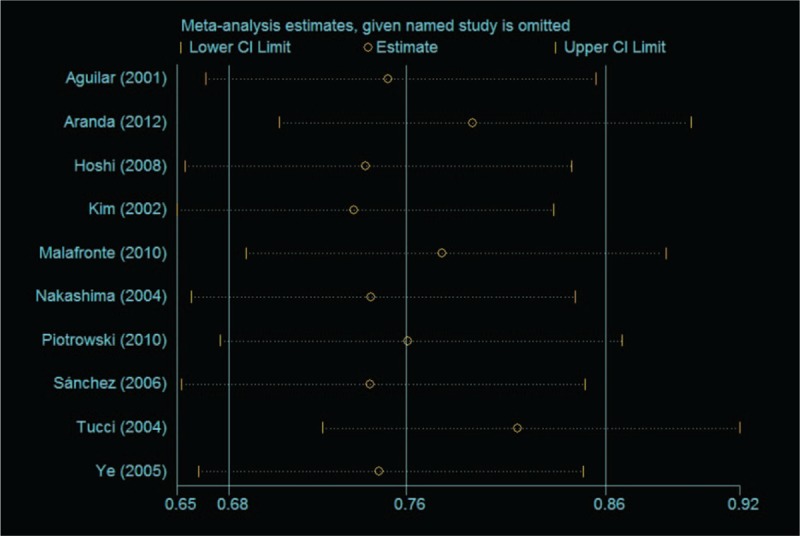
Sensibility analysis for the LN risk associated with MCP-1–2518 A/G polymorphism (A vs G). LN = lupus nephritis, MCP-1 = monocyte chemoattractant protein-1.

We used sensitivity analysis to estimate individual study's influence on the pooled OR, and the result of sensitivity analysis showed no other single study influenced the summary OR qualitatively, suggesting stability of the meta-analyses (Fig. 6).

### 3.4 Publication bias

**Figure 7:**
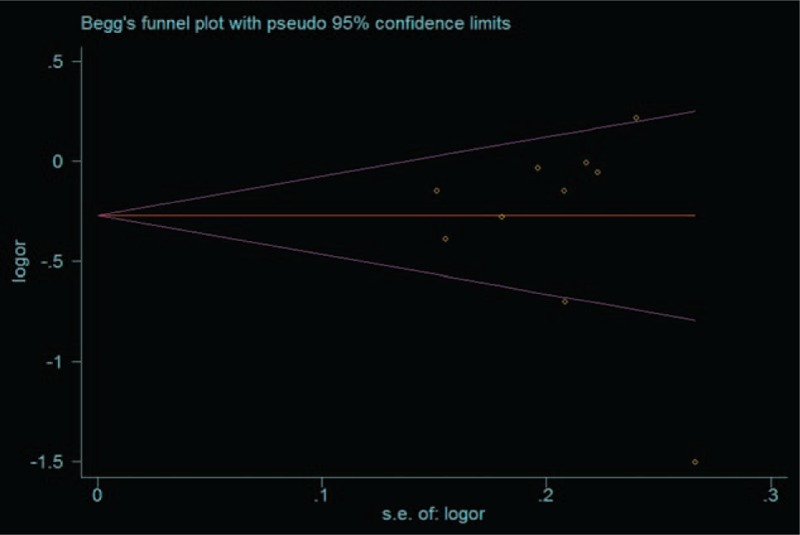
Funnel plot for publication bias of the meta-analysis of LN risk and MCP-1–2518 A/G polymorphism (A vs G). LN = lupus nephritis, MCP-1 = monocyte chemoattractant protein-1.

Funnel plots are shown in Fig. 7 for allele model. Arrangement of data points revealed evidence of symmetry which reflected that publication bias of the meta-analysis was not significant. Formal evaluation using Egger regression asymmetry tests for allele model and the result still did not show any evidence of publication bias (*t* = −0.66, *P* = .53).

## 4 Discussion

The present meta-analysis, including 961 LN cases and 1867 controls from 10 published case-control studies, showed that the MCP-1-2518 A/G was associated with LN risk. When stratified by different ethnicities, we found no significant association with European and Asian populations, which mainly occurred in the American population. This difference may be due to the genetic background of various ethnic populations and the environmental exposure. Further research on MCP-1 gene can better clarify the biological characteristics of LN, which can help us to detect, prevent, and treat LN.

Previous meta-analysis suggested that the polymorphism of MCP-1 gene was not associated with the incidence of LN, but which was associated with the susceptibility of LN in Caucasian population.^[18]^ The results from the present meta-analysis have a certain differences. The differences may be due to the publication of the latest case-control studies, but it is still more likely to be further confirmed based on larger population and more ethnicities studies. In many comparisons, there was heterogeneity, which brought the potential interference to the study. Although we minimized the likelihood by performing a careful search for published studies, using strict criteria for study inclusion, precise data extraction, and careful data analysis, significant between study heterogeneity existed in some comparisons. Then the subgroup analysis to a certain extent reduced the heterogeneity, but it was not entirely able to completely control the heterogeneity at a very low level. In the method of statistical analysis, we used the random-effect model to deal with the obvious heterogeneity (I^2^>50%, *P* < .1). Since the included studies were multicenters and regions, the heterogeneity of sources may come from the genetic, geographical environment, and living habits, etc.

The reasons for the difference found between the Caucasians and Asians population may be multifaceted. The difference may be related to the presence of other environmental factors and the distribution of the gene. LN is a result of SLE. A previous research^[18]^ found AA genotype might be a biomarker for the patients with SLE developing into LN, and the AA genotype was associated with the onset of SLE in Caucasians. Furthermore, a trend toward an association between A allele and LN risk was observed in Caucasians. The results of gene polymorphisms suggested the susceptibility of certain populations to LN, and the direct application is screening for this population. In addition, a number of articles reported the relationship between polymorphisms and LN treatment, including efficacy and side effects,^[19,20]^ and drug interactions.^[21]^ However, few studies have reported that MCP-1-2518 A/G polymorphism is involved in the therapeutic implications of LN.

The pathogenesis of LN has many aspects, and is regulated by many genes and signals. The analysis of a single nucleotide variation may not be able to fully elucidate the genetic association of LN, which may involve multiple genes. In addition, this study has the following limitations: First, the number of sample sizes included in the study was still not enough, it may cause some unstable results; Second, only English-language studies that were included in this meta-analysis might have led to publication bias, and the exclusion of unpublished data was generally associated with an overestimation of the true effect; Third, controls were not uniformly defined, while our result was based on unadjusted estimates.

In conclusion, our results suggested the MCP-1 gene-2518 A/G polymorphism was correlated with the presence of LN. Subgroup analysis found the genetic susceptibility was mainly associated with the American population, whereas there was no significant association between European and Asian populations. In future studies, more and larger case-control studies are needed to obtain further confirmation. Larger samples among different ethnicities, especially more sophisticated gene–gene and gene–environment interactions, should be considered in future studies, which should lead to better, comprehensive understanding of the association between MCP-1-2518 A/G polymorphism and LN risk.
